# Supplementary material for: RB dependent transcriptional regulation at mitotic centromeres preserves genome stability
Source: Life Sci Alliance. 2025 Dec 18;9(3):e202503433. doi: 10.26508/lsa.202503433 (PMC12715377; doi:10.26508/lsa.202503433)
Supplement: Supplementary file 1 [file LSA-2025-03433_TableS1.docx]

| **qPCR primer sequences** | | | |
| --- | --- | --- | --- |
| **Target** | | **Sequence** | **Source** |
| Centromere | Line-1 ORF1 | AAATGGTGCTGGGAAAACTG | Paul *et al.,* 2024 |
|  | Line-1 ORF2 | GTTTTGGACATGAAGTCCTTGC |  |
|  | D7Z1 | GTGGAGATATGGACCGCTTTAG | Contreras-Galindo *et al*, 2017 |
|  |  | CTCAGTCGTCACCAAGAGTTT |  |
|  | D17Z1 | GTGGAGATATGGACCGCTTTAG |  |
|  |  | CTCAGTCGTCACCAAGAGTTT |  |
|  | α-Sat1 | CATCACAAAGAAGTTTCTGAGAATGCT |  |
|  |  | TGCATTCAACTCACAGAGTTGAACCTTCC |  |
|  | α-Sat13/21 | TAGACAGAAGCATTCTCAGAAACT | Bosco *et al*, 2023 |
|  |  | TCCCGCTTCCAACGAAATCCTCCAAAC |  |
| Pericentromere | D7Z2 | CGACTTTGTGATGTGTGCATTC | Contreras-Galindo *et al.*, 2017 |
|  |  | CCTTATCCGCAATGGTCCTAAA |  |
|  | D17Z1b | TTTCGTAGGGTCTGCAAGTG |  |
|  |  | CCGACAATGCTTCTCTCTAGTT |  |
|  | K111 | AAGAGCACCAGGATGCTTAATGCC |  |
|  |  | AGTGACATCCCGCTTACCATGTGA |  |
| Gene | RB1 | TGGTGAATCATTCGGGCATT | This paper |
|  |  | GGTTTAGGAGGGTTGCTTCC |  |
|  | GAPDH | CGGAGTCAACGGATTTGGTC |  |
|  |  | CTTCCCGTTCTCAGCCTTGA |  |
|  | SNPRN | GCCAAATGAGTGAGGATGGT | Sanz *et al.,* 2019 |
|  |  | TCCTCTCTGCCTGACTCCAT |  |
|  | | | |
| **si and shRNA sequences** | | | |
| **Target** | | **Sequence** | **Source** |
| siScr#1 | | UGGUUUACAUGUCGACUAA | This paper |
| siScr#2 | | UGGUUUACAUGUUGUGUGA |  |
| siScr#3 | | UGGUUUACAUGUUUUCUGA |  |
| siScr#4 | | GGUUUACAUGUUUUCCUA |  |
| siRB#1 | | GAACAGGAGUGCACGGAUA | Manning *et al.*, 2014 |
| siRB#2 | | GGUUCAACUACGCGUGUAA |  |
| siRB#3 | | CAUUAAUGGUUCACCUCGA |  |
| siRB#4 | | CACCCAGCAGUUCGAUAU |  |
| shRB | | AGCAGTTCGATATCTACTGAAA | Zamalloa *et al.*, 2023 |

Supplemental Table 1: Primers and si/shRNA sequences used in this study.
